# Environmental Stress and the Deterministic Assembly of Bacterial Communities in Daqu: The Role of Amino Acid Content Fluctuations

**DOI:** 10.3390/foods14050725

**Published:** 2025-02-20

**Authors:** Zhihao Chen, Shaopei Tang, Xia Zhu, Guojun Zhu, Xiaoye Luo, Xiaodan Wang

**Affiliations:** 1 College of Liquor and Food Engineering, Guizhou University, Guiyang 550025, China; zhihaochen4990@163.com (Z.C.); zhuxia_2020@163.com (X.Z.); luoxiaoye20172044@126.com (X.L.); 2 Kweichow Zhen Distillery Co., Ltd., Zunyi 563000, China; tsp668@zjld.com (S.T.); zgj588@vats.com.cn (G.Z.)

**Keywords:** daqu, microbial assembly, driving factors, nutrient fluctuation, fermentation, metabolic pathways

## Abstract

Bacterial communities are highly susceptible to fluctuations in amino acid content. To investigate the response of microbial communities in daqu to environmental perturbations, we employed high-throughput sequencing and statistical analyses. Samples were collected from two workshops (A and B) at distinct stages of daqu fermentation and storage. Our analysis, using the β-nearest taxon index (βNTI), revealed that fungal community assembly is shaped by both stochastic and deterministic processes. In contrast, bacterial communities exhibited a shift towards deterministic assembly under environmental stress, with fluctuations in amino acid content being a primary driver. Notably, communities with active amino acid metabolism displayed a greater involvement of stochastic processes and harbored a higher number of bacterial keystone taxa, which contributed to the stability of microbial networks. This study provides novel insights into the complex interplay between microbial communities and their environment in the context of daqu.

## 1. Introduction

Chinese baijiu, renowned for its rich history and cultural importance, ranks among the world’s top six distilled spirits, owing to its unique solid-state fermentation process and substantial market impact [1]. Baijiu, a traditional distilled spirit, is crafted from sorghum and daqu, with the latter acting as the primary fermentation agent. Daqu introduces diverse functional microbial communities that play a significant role in the accumulation of ethanol and flavor compounds during baijiu fermentation [2,3,4,5]. The colonization success in ecological niches is determined by both abiotic and biotic factors. Abiotic factors include moisture, temperature, and nutrient availability, while biotic factors encompass microbial cooperation and competition. These factors dynamically evolve throughout the community assembly process [6,7]. Meanwhile, numerous models and experiments have demonstrated that the strength of cooperation and competition can be influenced by the concentration of environmental nutrients [8]. Nutrient fluctuations can significantly affect microbial community assembly and metabolic functions [9]. However, this concept has not been extensively applied to daqu during fermentation and storage.

Daqu undergoes spontaneous fermentation in an open environment, with microorganisms primarily utilizing carbon compounds and amino acids as essential nutrients [9]. This metabolic process is marked by the upregulation of genes associated with carbon and amino acid metabolism [10]. Fungi dominate carbon metabolism in daqu, regulating this process through various extracellular enzymes [10,11]. Bacterial amino acid metabolism can induce microecological differentiation, thereby affecting the appearance and overall quality of daqu [12,13]. Understanding the ecological and evolutionary constraints of daqu is crucial for optimizing fermentation. It is essential to determine how trophic selection pressures affect microbial assemblages and community stability.

Current research on daqu microecology primarily investigates species and metabolite succession, examining their relationships with raw materials, environmental factors, interventions, and product quality [5,14,15,16,17,18]. Nevertheless, microbial community stability remains understudied. Stochastic and deterministic processes combine to shape the microbial communities in fermented foods [19]. Numerous studies have investigated variations in community assembly among different types of daqu [20,21,22]. However, there is a significant knowledge gap concerning community assembly during daqu fermentation and storage.

This study analyzed daqu samples from diverse environments during fermentation and storage stages. We utilized Illumina HiSeq sequencing and multiple statistical methods to investigate microbial community dynamics in daqu. Microbial communities from different nutrient environments were compared using neutral community models and network analyses. Additionally, we also explored correlations between keystone taxa and environmental parameters. Lastly, PICRUSt2 was employed to predict differential metabolic pathways across sample groups.

## 2. Materials and Methods

### 2.1. Sample Collection

Samples were collected from two workshops (A and B) at Kweichow Zhen Distillery Co., Ltd. (Zunyi, China). We randomly selected three fermentation chambers from each workshop to conduct fermentation experiments simultaneously under the same external environment. The collection period (in summer) covered the initial 40 days of fermentation in the fermentation room (8 m × 5 m × 3 m), followed by 90 days of storage in the warehouse (15 m × 10 m × 3 m). The sampling time points were set at 0 d, 10 d, 20 d, and 40 d during the fermentation phase and at 30 d and 90 d during the storage phase. The corresponding samples were labeled as F00, F10, F20, F40, S30, and S90. At each sampling time point, five daqu bricks each were randomly chosen from the top, middle, and bottom layers of the stack. These 15 bricks were then crushed and mixed evenly. The mixture was placed in a sterile bag, constituting a single daqu sample. The samples were promptly analyzed for their physicochemical properties and stored at −80 °C for subsequent DNA extraction. A total of 540 daqu bricks were collected, resulting in 36 bags of daqu samples.

### 2.2. Determination of Physicochemical Indices

The moisture, total acidity, and reducing sugar content of daqu samples were tested using the Chinese daqu brewing method (QB/T4257-2011 [23]) as the experimental standard. The core temperature of the daqu was measured using a digital stainless steel probe thermometer (WST-411; SUJIE, Yancheng, China). This thermometer was inserted into the daqu bricks, and the measurements were repeated five times in each fermentation room to ensure accuracy.

### 2.3. Quantitative Analysis of Carbohydrates and Amino Acids

An automatic amino acid analyzer was used to analyze free amino acids in daqu (LA8080 Hitachi, Tokyo, Japan), following the GB-T 18246-2019 [24] standard for amino acid determination in feed. Identification was based on retention time, and quantification was achieved using the external standard method.

### 2.4. Volatile Metabolites Analysis

Volatile organic compounds (VOCs) were determined by HS-SPME-GC-MS (Agilent 8890-7000D; Agilent Technologies, Santa Clara, CA, USA) using a DB-Wax column (30 m × 250 μm × 0.25 μm). The instrument was operated with a He flow rate of 2.25 mL/min and a N_2_ flow rate of 1.5 mL/min. The initial temperature was set to 40 °C and held for 3 min, followed by an increase to 230 °C at a rate of 4 °C/min, where it was held for an additional 3 min. The inlet port temperature was maintained at 250 °C, and the samples were injected in splitless mode. An electron ionization (EI) ion source with an electron energy of 70 eV, an ion source temperature of 230 °C, and an interface temperature of 250 °C was employed. The acquisition mode was SCAN, covering a mass scanning range of *m*/*z* 35–400 amu. VOC identification was achieved by comparing experimental mass spectra with the NIST 20 MS spectral database [25]. 2-Octanol served as an internal standard, and VOC content was quantified based on peak intensity.

### 2.5. DNA Extraction, Amplification, and Sequencing

The PCR primer was designed to target regions of the 16S and ITS2 rDNA genes, which are conserved across species but exhibit variability. After 35 PCR cycles, sequencing adapters and barcodes were incorporated. The PCR products were analyzed by 1.5% agarose gel electrophoresis. Target fragments were recovered using the AxyPrep PCR Cleanup Kit (Axygen, Hangzhou, China) and further purified using the Quant-iT PicoGreen dsDNA Assay Kit (Jinpan, Shanghai, China). Library quantification was conducted using the Promega QuantiFluor fluorescence quantification system. The pooled library was then loaded onto the Illumina platform for paired-end sequencing (2 × 250 bp). Paired-end reads were merged using FLASH (v1.2.8) for 16S and PEAR (v0.9.6) for ITS2 sequences. Raw reads underwent quality filtering following the fqtrim (v0.94) protocol to obtain high-quality clean tags. Chimeric sequences were removed using Vsearch software (v2.3.4). After dereplication using DADA2, feature tables and sequences were obtained.

### 2.6. Ecological Community Assembly Analysis

The relative contributions of stochastic and deterministic processes in daqu fermentation were assessed by calculating βNTI. βNTI quantifies the degree of community assembly driven by deterministic or stochastic processes. We calculated βNTI using the ape (v5.7-1) and picante (v1.8.2) packages in R (v4.3.2) [26,27]. Specifically, |βNTI| > 2 indicates that the community is controlled by deterministic assembly, which influences community succession; when βNTI > 2, it indicates a homogeneous selection process, while βNTI < −2 indicates a deterministic selection process. Conversely, |βNTI| < 2 suggests that stochastic processes are dominant.

To further assess the importance of stochastic assembly in community structure, we applied the Sloan neutral community model [28]. We used a modified version of the R code mentioned in [29]. In this model, R^2^ represents the overall goodness of fit of the neutral community model. N represents the metacommunity size, defined as the total abundance of all amplicon sequence variants (ASVs) in each sample. m quantifies the community-level migration rate, with lower values indicating more restricted species dispersal and higher values indicating less restricted dispersal. Nm, the product of metacommunity size (N) and mobility (m), estimates inter-community dispersal and determines the correlation between species occurrence frequency and relative abundance.

### 2.7. Statistical Analyses

A one-way analysis of variance (ANOVA) and the Tukey test were performed using IBM SPSS 22 (IBM, New York, NY, USA). Principal coordinate analysis (PCoA) was conducted on microbial data from both groups using the vegan package (v2.6-4) in R (v4.3.2). Subsequently, permutational multivariate analysis of variance (PERMANOVA) was performed on the data between groups using the pairwiseAdonis package (v0.4.1). Redundancy analyses were also performed using the vegan package (v2.6-4) in R (v4.3.2) to investigate the correlation between microbes and environmental factors. Spearman’s rank correlation of microbial ASVs (mean abundance > 0.01%) was calculated using the Hmisc package (v5.1-1) in R (v4.3.2). Significant correlations (*p* < 0.05, false discovery rate correction and |ρ| > 0.6) were used to characterize node topology in ggplot2 (v3.4.4) and to construct microbial networks in Gephi 10.0 (Web Atlas, Paris, France).

## 3. Results and Discussion

### 3.1. Sequence Quality Control

A total of 2,640,371 valid tags from the V3 to V4 region of the 16S rRNA gene and 2,703,320 valid tags from the internal transcribed spacer (ITS) region were obtained from 26 samples. After dereplication using DADA2, a total of 5069 bacterial ASVs and 971 fungal ASVs were used in subsequent analyses. Good’s coverage index for each Daqu sample exceeded 99.8%, indicating that the sequencing results are highly reliable.

### 3.2. Temporal Changes in Fermentation Parameters and Metabolites

**Figure 1:**
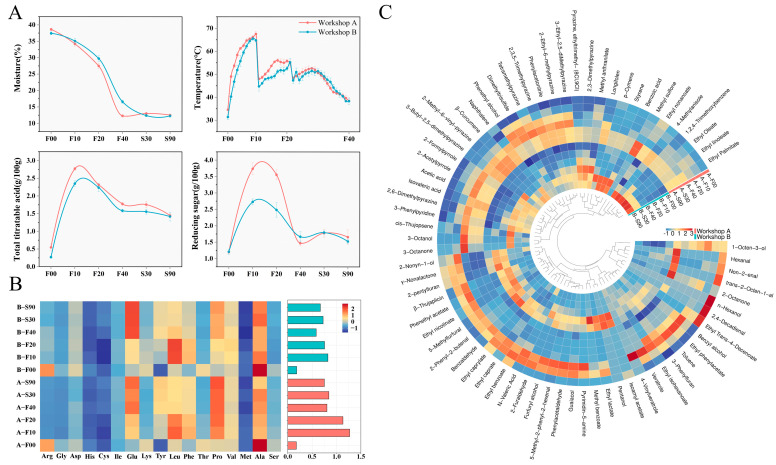
Temporal variations in fermentation parameters (**A**), encompassing changes in Daqu core temperature, moisture content, total titratable acidity, and reducing sugar levels. Heatmap and histogram depicting amino acid content (**B**) in Workshop A and Workshop B throughout fermentation and storage periods, with values expressed in g/100 g. A heatmap illustrating volatile metabolite profiles (**C**) in both workshops across the fermentation and storage stages. The color scale signifies the normalized abundance of each amino acid or volatile metabolite, denoted as the Z-score, where red and blue denote high and low abundances, respectively. F, fermentation; S, storage. The numerals following these letters indicate the respective days.

Daqu fermentation occurs in an open environment, where environmental factors significantly influence the dynamic succession of microbial communities, resulting in variations in metabolism and functional attributes [30]. As shown in Figure 1A, the core temperatures of daqu in both workshops followed similar trends throughout the fermentation process. After entering the warehouse, the core temperatures of the raw daqu increased rapidly, with Workshop A showing a higher rate of temperature increase than Workshop B during the first 7 days. Following traditional practices, turnovers were performed on the 10th (65 ± 1 °C) and 20th (53 ± 3 °C) days of fermentation to regulate temperatures and ensure optimal daqu fermentation. Each turnover helped regulate the microbial community structure [18]. Notably, Workshop A showed a greater warming capacity after each turnover compared to Workshop B.

During the entire production period, the water content of daqu decreased gradually from 38.03 ± 0.73% at the beginning to 12.43 ± 0.34% at maturity. A noticeable difference in water content between the two workshops was observed during F20 and F40, with Workshop A showing significantly lower levels than Workshop B. This difference can be attributed to the different warming rates between the workshops. The Maillard reaction in daqu under high-temperature conditions tends to produce high-quality daqu with lower water content [31].

Reducing sugar levels decreased gradually after the initial turnover and were significantly higher (*p* < 0.05) in Workshop A (3.64 ± 0.19 g/100 g) than in Workshop B (2.61 ± 0.21 g/100 g) during fermentation. This suggests that the microorganisms in Workshop A may have been more metabolically active. Reducing sugar serves not only as a primary nutrient source for microorganisms in daqu but also as a precursor to crucial flavor compounds formed via the Maillard reaction [32].

Acidic environments result from the accumulation of organic acids produced by acid-producing microorganisms, which contributes to the formation of alcohols, esters, and aldehydes [33]. A significant difference in total acid content was observed between the two workshops, with both peaking on day 10 and then declining gradually. Previous research has shown that high-quality daqu tends to have higher acidity [34]. Insufficient acid-producing microorganisms can lead to incomplete fermentation, which can compromise daqu quality [35].

The total amino acid content was consistently higher in Workshop A (0.96 ± 0.20 g/100 g) than in Workshop B (0.71 ± 0.08 g/100 g) throughout both the fermentation and storage periods (*p* < 0.05) (Figure 1B). Significant differences were also observed in the levels of lysine, leucine, and valine, with Workshop A showing higher concentrations than Workshop B (*p* < 0.05). Amino acids are essential nutrients that can influence microbial growth, fermentation kinetics [9], and the synthesis of flavor compounds [36].

A total of 72 volatile metabolites were identified (Figure 1C), of which 14 substances showed statistically significant differences between the two workshops. Workshop A showed significantly higher concentrations of 1-Octen-3-ol, ethyl isohexanoate, dimethyltrisulfide, ethyl benzoate, N-valeric acid, benzyl alcohol, 2,3,5-trimethylpyrazine, tetramethylpyrazine, and phenethyl alcohol than Workshop B (*p* < 0.05). In contrast, Workshop B showed significantly higher levels of isovaleric acid, pyrazine, ethyltrimethyl- (8CI, 9CI), 2,3-dimethylpyrazine, pentanol, and benzoic acid. Notably, pentanol and benzoic acid were present at significantly higher concentrations in the Workshop B samples than in those from Workshop A (*p* < 0.05).

### 3.3. Microbial Diversity and Dynamics

**Figure 2:**
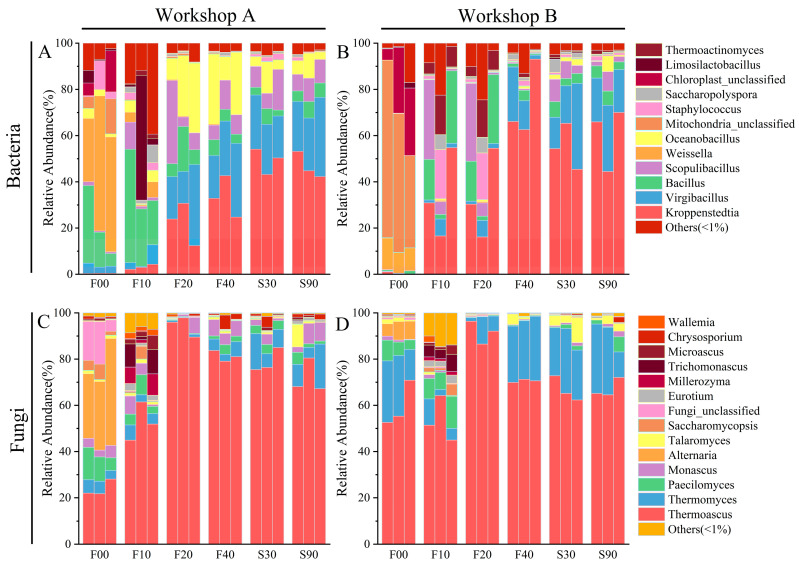
Dynamics of relative abundance of bacteria (**A**,**B**) and fungi (**C**,**D**) in workshops A and B. Only genera that had an average abundance of >1% are indicated.

The microbial communities of daqu samples from two workshops were analyzed using the Illumina HiSeq platform. Initially, raw daqu was dominated by *Weissella* (45.21%) and *Bacillus* (18.02%). On day 10 of fermentation, Workshop A exhibited the highest *Bacillus* content (31.03%) (Figure 2A), while Workshop B showed the highest *Kroppenstedtia* content (34.05%) (Figure 2B). At F20, the five most abundant genera in Workshop A were *Virgibacillus* (22.4%), *Kroppenstedtia* (22.3%), *Oceanobacillus* (21.82%), *Scopulibacillus* (15.81%), and *Bacillus* (16%). In Workshop B, the top three dominant genera were *Kroppenstedtia* (33.63%), *Bacillus* (16.32%), and *Scopulibacillus* (13.47%). From F40 to S30, *Kroppenstedtia* was the most prevalent bacterium (53.04%). However, *Oceanobacillus* (12.01%) and *Scopulibacillus* (9.81%) were more abundant in Workshop A than in Workshop B. Throughout the daqu production process, the dominant bacteria transformed from early *Bacillus* to mature *Kroppenstedtia* and *Virgibacillus*. Previous studies have shown that *Bacillus* is strongly correlated with the production of esters, amino acids and their derivatives, and sugars and their derivatives [37]. Both *Bacillus* and *Kroppenstedtia* exhibit high protease activities, enriching S-(hydroxymethyl)glutamate and S-(formylmethyl)glutamate in daqu, and are closely associated with the synthesis of amino acids, organic acids, and other flavor substances [38,39].

In the ITS1 region, *Thermoascus* (67.69%) played a dominant role throughout the fermentation process, potentially being involved in encoding enzymes related to saccharification and ethanol fermentation [40]. The samples from Workshop B had significantly higher levels of *Thermomyces* (24.28%) (Figure 2D) compared to those from Workshop A (7.25%) (Figure 2C). Both *Thermomyces* and *Thermoascus* are the primary fungi involved in encoding enzymes in daqu [41]. Workshop A detected higher levels of *Monascus*, a fungus positively correlated with glucoamylase in daqu, compared to Workshop B [42].

**Figure 3:**
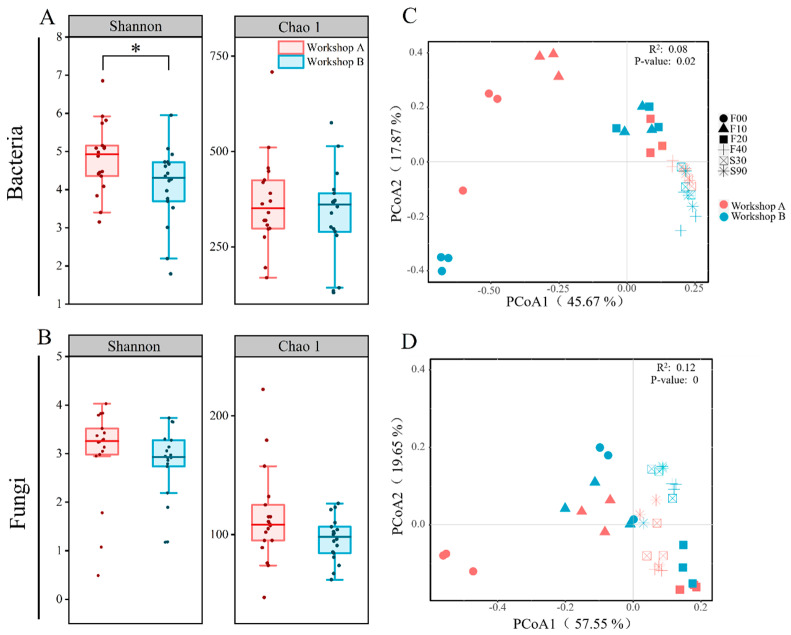
Box and whisker plots showing variance in alpha diversity indices—Shannon and Chao1—of bacterial communities (**A**) and fungal communities (**B**), *: *p* < 0.05. Principal coordinate analysis (PCoA) based on the Bray–Curtis dissimilarity matrix for microbial communities: (**C**) bacteria and (**D**) fungi.

Principal Coordinate Analysis (PCoA) using the Bray–Curtis distance matrix at the genus level demonstrated notable dispersion of bacterial communities during fermentation and clustering during the storage phase (S30–S90) in both workshops (Figure 3C). In contrast, fungal communities remained dispersed throughout the fermentation phase (Figure 3D). Permutational multivariate analysis of variance (PERMANOVA) revealed significant diversity in bacterial (R^2^ = 7.97%, *p* = 0.033) and fungal (R^2^ = 11.83%, *p* = 0.006) communities between the two workshops. However, no significant difference was observed in bacterial communities during the storage phase (R^2^ = 20.62%, *p* = 0.085).

Microbial abundance did not differ significantly between the two workshops (Figure 3B), but the Shannon index of Workshop A was significantly higher than that of Workshop B (Figure 3A), indicating greater bacterial community diversity in Workshop A.

### 3.4. Community Assembly Patterns and Correlation Analysis

**Figure 4:**
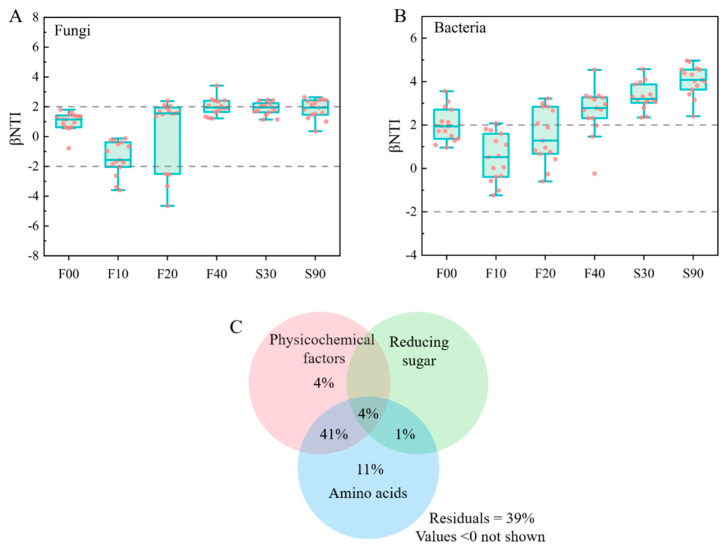
βNTI values of bacterial (**A**) and fungal (**B**) communities, when |βNTI| is greater than 2, it signifies deterministic assembly; when |βNTI| is less than 2, it indicates stochastic assembly. Variation partitioning analysis (VPA) shows the percentage contribution of external factors to the bacterial communities (**C**).

On the basis of the framework developed by Stegen [43], βNTI was used to quantify how microbial communities are assembled during daqu fermentation. During the early stages of fermentation, on the 10th and 20th days, the bacterial community was predominantly influenced by stochastic assembly processes (93.33% and 66.67%, respectively). However, as fermentation progressed, a gradual shift was observed towards deterministic processes, with variable selection ultimately determining the composition of the mature bacterial community in daqu (Figure 4A). In contrast, fungal community assembly was largely stochastic (70%) throughout the fermentation period (F00–F40), while during storage, it was governed by a combination of deterministic and stochastic processes (53.33% and 46.67%, respectively) (Figure 4B).

According to ecological niche theory, microbial communities are shaped both by abiotic factors (such as temperature, nutrients, and pH) and biotic factors. Our findings indicate that bacterial communities present during fermentation are primarily shaped by deterministic processes, suggesting that the internal ecology of daqu selects for bacteria with distinct preferences and adaptations to varying growth environments. To assess the contribution of each factor to the variation in bacterial β-diversity during fermentation, we performed a variance partition analysis (VPA). The results revealed that amino acids alone accounted for 11% of the variation in the bacterial community (Figure 4C), and when combined with physicochemical factors (temperature, moisture, and total acid), they explained 41% of the variation. These findings suggest that amino acid content is a crucial driver of deterministic bacterial community assembly.

Previous studies have shown that microbial communities are more prone to stochastic processes when biomass and population size are relatively low [44]. In the initial stages of microbial community assembly in daqu, the environment is abundant in nutrients and spatial niches, allowing for random survival, growth, and dispersal of microorganisms, resulting in a stochastic assembly process. As the community assembly progresses, the limited availability of space and nutrients (carbohydrates and amino acids) leads to increased environmental stress. Bacteria exhibit greater sensitivity to environmental changes compared to fungi, leading to a transition in the bacterial community towards a deterministic assembly process. Here, heterogeneous selection (HeS) (βNTI > 2) plays a crucial role. Based on HeS, environmental changes contribute to high β-diversity by selecting dominant species from local species pools [45]. Our results indicate that amino acids, moisture, temperature, and total acid have the potential to induce shifts in bacterial community structure and assembly processes, with amino acids exerting the most prominent influence.

A significant proportion of bacterial species are unable to synthesize the essential amino acids required for their growth, necessitating their acquisition from the surrounding environment. This scenario favors the survival of amino acid auxotrophs in environments rich in these compounds, as evidenced by Ramoneda [46]. Fermented foods and the human gut are notable examples of ecosystems abundant in amino acids, harboring many amino acid auxotrophies [47,48,49]. The rationale is straightforward: when microorganisms have uninterrupted access to an abundant and inexhaustible nutrient source, there is no incentive for them to synthesize amino acids de novo, as they can easily obtain them from the environment. This may explain the higher diversity of bacterial communities observed in the Workshop A samples, which had a higher amino acid content, compared to those from Workshop B. Such an amino acid cross-feeding mechanism may contribute to ecological stability [50]. Therefore, further research is necessary to confirm the stability of the microbial communities in both workshops.

Amino acids serve as crucial nutrients for microorganisms, supporting bacterial growth and fermentation kinetics [9]. It is reasonable to hypothesize that the abundant amino acid content during the fermentation process reduces environmental stresses and promotes the enrichment of bacteria in daqu, as well as the survival of amino acid auxotrophs. Additionally, in ecosystems where a wide range of organisms can grow freely, stochastic assembly is often more prevalent.

### 3.5. Fit to the Neutral Model of Community Assembly

**Figure 5:**
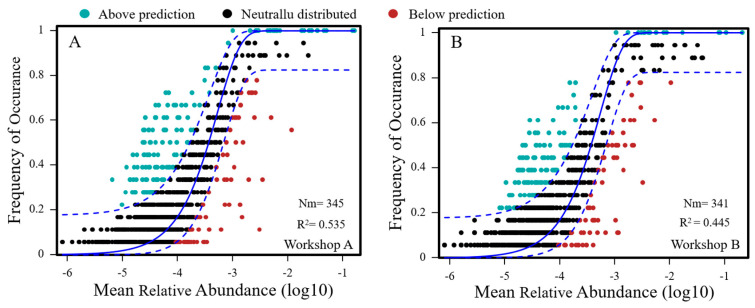
Neutral community model of Workshop A (**A**) and Workshop B (**B**); the solid blue line indicates the best fit of the model, and the dashed blue line indicates the 95% confidence interval around the model predictions. ASVs that occurred more frequently than predicted by the model are shown in green, while those that occurred less frequently than predicted by the model are shown in red.

To quantify the impact of stochastic processes during daqu fermentation on assembly differences between the two sets of workshop samples with different amino acid contents, we used the neutral community model (NCM) proposed by Sloan [28] (Figure 5). As a result of the NCM, the relationship between the frequency and relative abundance of amplicon sequence variants (ASVs) was effectively estimated. The R^2^ value, indicating the overall goodness of fit of the NCM, was 0.535 for Workshop A and 0.455 for Workshop B. Having a higher R2 value indicates that stochastic processes are more influential on community structure. The Nm values showed that species dispersal was slightly higher in Workshop A (345) than in Workshop B (341).

### 3.6. Complexity and Stability of Microbial Networks

**Figure 6:**
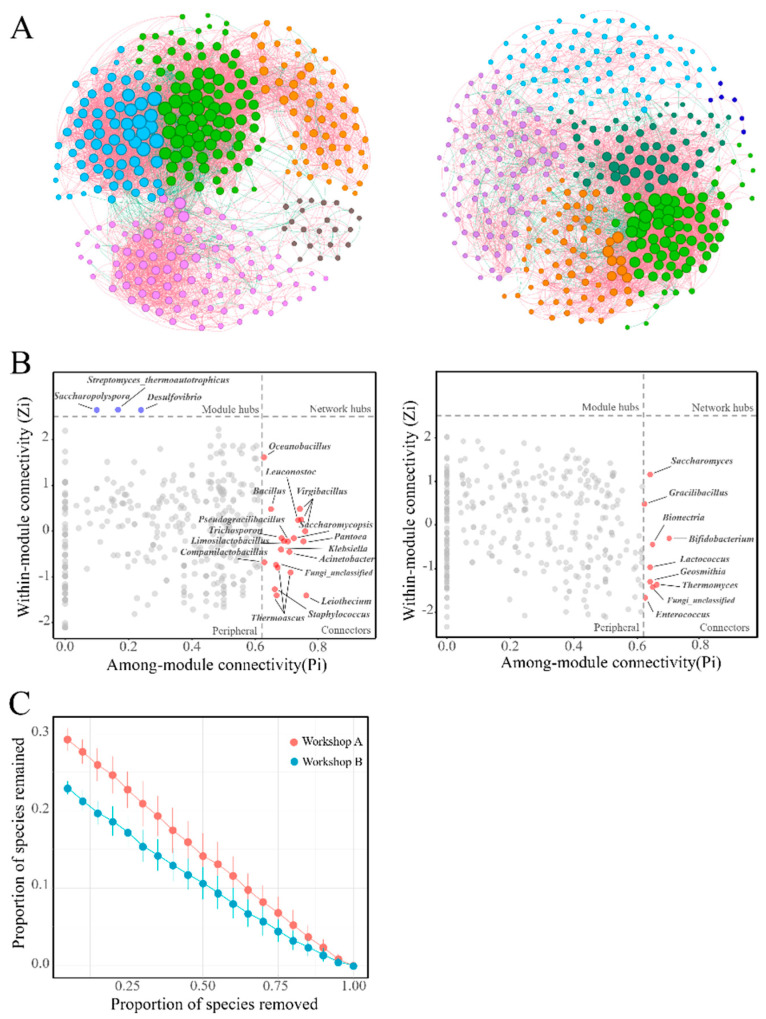
Networks of cooccurrence patterns (**A**) were constructed by selecting ASVs that had a relative abundance greater than 0.01% and occurred in more than 30% of the samples, where the color of the nodes depends on the module, and the size depends on the degree of connectivity. Red edges indicate a positive correlation between nodes, and a green color represents a negative correlation. (**B**) We calculated the within-module connectivity (Zi) and among-module connectivity (Pi) of the nodes to determine the node properties. We also performed a robustness analysis on the microbial communities of the two workshops (**C**)—the more taxa remaining after randomly removing taxa from the network, the more stable the community.

To compare the complexities and stability of the microbial networks, two networks were constructed from the samples (*n* = 36) of the two workshops. Workshop A has 361 nodes and 4889 edges, while Workshop B has 339 nodes and 6647 edges (Figure 6A). Workshop B has five large modules (each with at least five nodes), while Workshop A has six such modules. However, Workshop B shows higher modularity (0.469 vs. 0.418) than Workshop A, indicating greater connectivity among the nodes within its modules. Additionally, the percentage of negative correlation in Workshop A (16.92%) is higher than in Workshop B (14.41%).

Node topological roles were evaluated using within-module connectivity (Zi) and among-module connectivity (Pi) [51], classifying them into peripherals, module hubs, network hubs, and connectors [52]. In Workshop A, 3 module hubs and 20 connectors were identified, totaling 23 keystone nodes. In contrast, only 9 connectors were found in Workshop B (Figure 6B). The proportion of bacterial keystone nodes in Workshop A (69.57%) was higher than in Workshop B (44.44%).

To evaluate the stability of microbial networks, species extinction was simulated by randomly eliminating a predetermined percentage of nodes [53]. The results showed that the microbial network of Workshop A was more robust (with less connectivity loss) than that of Workshop B (Figure 6C).

The topology of the microbial co-occurrence network in Daqu was influenced by resource availability and species diversity. Given the crucial roles of module hubs and connectors in network topology, they were identified as keystone taxa [52]. More keystone taxa were found in the nutrient-rich Workshop A samples than in the daqu samples from Workshop B. It is hypothesized that keystone taxa, acting as pivotal nodes, have unique metabolic attributes that may contribute to ecosystem stability. The absence of these taxa could lead to module and network disintegration [54,55]. The robustness analysis of microorganisms in both workshops further supported this conclusion.

Furthermore, Workshop A, with ample nutrient substrate, facilitated microbial growth, reproduction, and random death, leading to a stochastic assembly process that increased species diversity and intensified interspecies competition. This competitive dynamic resulted in a higher proportion of negative interactions in Workshop A than in Workshop B, contributing to its greater stability. Positive interactions indicate species occupying similar ecological niches. Under environmental stress, a decline in the abundance of one species could indirectly reduce the abundance of related species, jeopardizing community stability [56]. In contrast, the presence of negative correlations mitigated these disturbances [57].

### 3.7. Exploring for Abiotic Factors Driving the Keystone Node Metabolism

**Figure 7:**
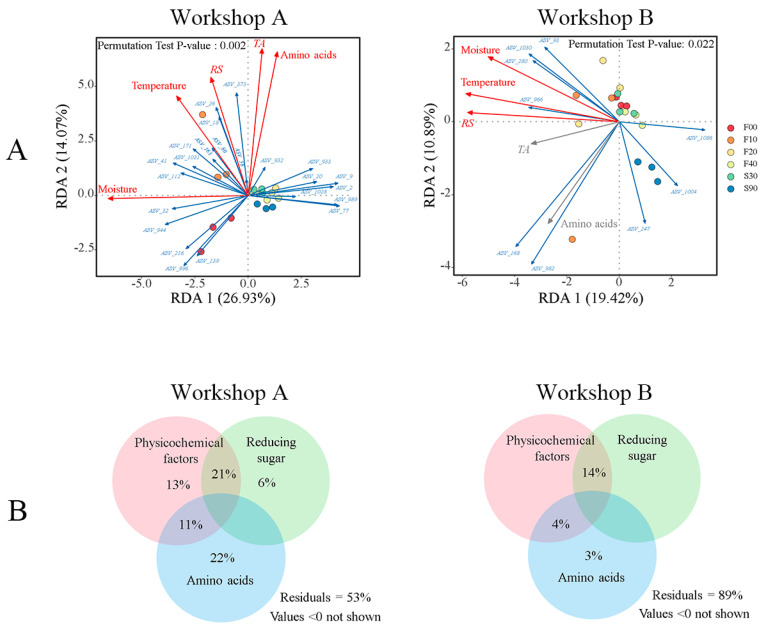
Redundancy analysis (RDA) plots showing the weights and orientation of the keystone taxa of the two workshops and environmental parameters (**A**). Variation partitioning analysis (VPA) showed the percentage contribution of external factors to the keystone taxa of the two workshops (**B**).

Redundancy analysis was conducted to examine the correlation between keystone nodes and environmental factors, as well as nutritional conditions (Figure 7A). In Workshop A, amino acids were positively correlated with 5 fungal ASVs and 12 bacterial ASVs (R^2^ = 71.83%, *p* = 0.001), representing 77.27% of the total. Acidity (R^2^ = 71.39%, *p* = 0.001) was positively correlated with 4 fungal ASVs and 11 bacterial ASVs, accounting for 68.2% of the total. Moisture (R^2^ = 70.32%, *p* = 0.001) was positively correlated with 63.6% of the nodes. Reducing sugars (R^2^ = 47.87%, *p* = 0.01) and temperature (R^2^ = 46.7%, *p* = 0.008) were positively correlated with 13 and 12 keystone nodes, respectively. In Workshop B, moisture (R^2^ = 45.53%, *p* = 0.001), reducing sugars (R^2^ = 57.9%, *p* = 0.002), and temperature (R^2^ = 59.51%, *p* = 0.001) were positively correlated with the same six keystone nodes. Variance partitioning analysis (Figure 7B) further revealed that amino acids alone explained 22% of the variation in Workshop A and 3% in Workshop B, indicating that amino acids are key drivers in the formation of keystone taxa.

### 3.8. Prediction of Metabolic Pathways

**Figure 8:**
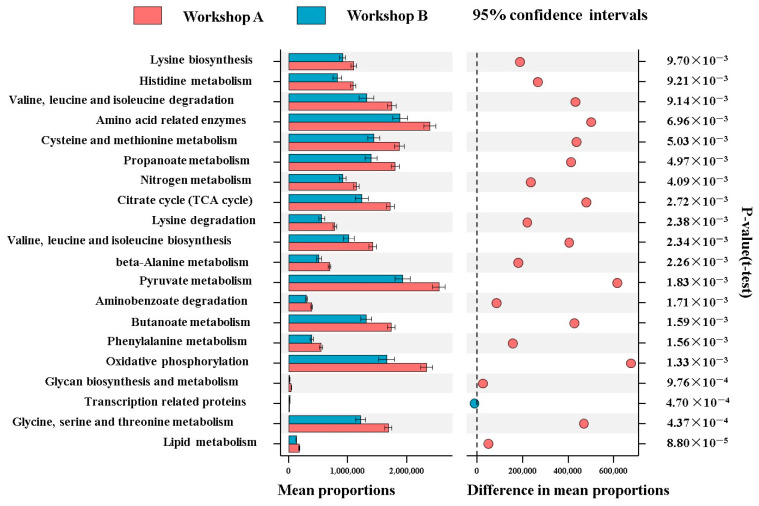
Comparison and prediction of metabolic pathways for different workshops. Points to the right of the dashed line indicate pathways where the mean abundance is higher in Workshop A (colored in Workshop A’s designated color). Points to the left of the dashed line indicate pathways where the mean abundance is higher in Workshop B (colored in Workshop B’s color). The distance of a point from the dashed line reflects the magnitude of the difference in mean abundance between the two workshops. Larger distances correspond to greater disparities.

The pathway prediction results are presented in a graph (Figure 8). Thirty different KEGG level 3 pathways were predicted and screened (*p* < 0.05).

Compared to Workshop B, amino acid metabolism (including methionine, phenylalanine, isoleucine, glycine, and serine) was more active in Workshop A, explaining the difference in amino acid content between the two workshops. Differences in amino acid metabolism have been linked to daqu differentiation, leading to variations in other metabolites [13,58]. Notably, pyruvate metabolism was significantly higher in Workshop A than in Workshop B (*p* < 0.05). Pyruvate is a crucial precursor for amino acid biosynthesis [59], indicating that Workshop A has a greater capacity to accumulate amino acids.

Additionally, several differentially expressed genes were found in pathways related to nitrogen metabolism, pyruvate metabolism, glycolysis/gluconeogenesis, the citrate cycle, oxidative phosphorylation, propanoate metabolism, butanoate metabolism, and lipid metabolism. These pathways expressed significantly more genes in Workshop A than in Workshop B (*p* < 0.05), indicating significant differences in carbohydrate and energy metabolism between the two workshops. The energy released from carbohydrate catabolism is essential for microbial metabolic activity and growth. Microorganisms in Workshop A can participate in metabolism more efficiently. The abundance of energy sources and metabolic strategies in Workshop A contributes to maintaining microbial diversity [60].

## 4. Conclusions

In conclusion, fluctuations in amino acid content and metabolic intensity significantly influence bacterial community assembly patterns within daqu. Active amino acid metabolism promotes the establishment of more keystone taxa, enhancing species diversity and community stability. Conversely, declining amino acid concentrations lead to bacterial communities governed by deterministic assembly processes. This insight will aid in designing more efficient daqu fermentation systems by manipulating community structure and metabolic composition. Furthermore, regarding future research, we aim to isolate amino acid-rich strains during daqu fermentation and investigate the impact of functional microorganisms on daqu quality.

## Data Availability

The original contributions presented in this study are included in the article. Further inquiries can be directed to the corresponding author.

## References

[1] Wang L. (2022). Research Trends in Jiang-Flavor Baijiu Fermentation: From Fermentation Microecology to Environmental Ecology. J. Food Sci..

[2] Huang Y., Yi Z., Jin Y., Huang M., He K., Liu D., Luo H., Zhao D., He H., Fang Y. (2017). Metatranscriptomics Reveals the Functions and Enzyme Profiles of the Microbial Community in Chinese Nong-Flavor Liquor Starter. Front. Microbiol..

[3] Wang B., Wu Q., Xu Y., Sun B. (2020). Synergistic Effect of Multiple Saccharifying Enzymes on Alcoholic Fermentation for Chinese Baijiu Production. Appl. Environ. Microbiol..

[4] Wu Q., Zhu Y., Fang C., Wijffels R.H., Xu Y. (2021). Can We Control Microbiota in Spontaneous Food Fermentation?—Chinese Liquor as a Case Example. Trends Food Sci. Technol..

[5] Yang Y., Niu M.-S., Yu H., Shi W., Chai L.-J., Lu Z.-M., Liu X.-T., Shen C.-H., Xu Z.-H., Wang S.-T. (2024). Exploring the Contribution of Temperature-Adapted Microbiota to Enzyme Profile of Saccharification in *Daqu* Using Metagenomics and Metaproteomics. LWT.

[6] Fukami T. (2015). Historical Contingency in Community Assembly: Integrating Niches, Species Pools, and Priority Effects. Annu. Rev. Ecol. Evol. Syst..

[7] Gralka M., Szabo R., Stocker R., Cordero O.X. (2020). Trophic Interactions and the Drivers of Microbial Community Assembly. Curr. Biol..

[8] Christoph R., Julien B., Gore J. (2020). Strength of Species Interactions Determines Biodiversity and Stability in Microbial Communities. Nat. Ecol. Evol..

[9] Wei J., Lu J., Nie Y., Li C., Du H., Xu Y. (2023). Amino Acids Drive the Deterministic Assembly Process of Fungal Community and Affect the Flavor Metabolites in Baijiu Fermentation. Microbiol. Spectr..

[10] Zhang Y.-T., Deng Y.-K., Zou Y.-F., Han B.-L., Pu J.-Z., Rao J.-Q., Huang D., Luo H.-B. (2022). Linking Microbial Functional Gene Abundance and Daqu Extracellular Enzyme Activity: Implications for Carbon Metabolism during Fermentation. Foods.

[11] Zhu Q., Chen L., Peng Z., Zhang Q., Huang W., Yang F., Du G., Zhang J., Wang L. (2023). The Differences in Carbohydrate Utilization Ability between Six Rounds of Sauce-Flavor Daqu. Food Res. Int..

[12] Yang L., Fan W., Xu Y. (2023). Chameleon-like Microbes Promote Microecological Differentiation of Daqu. Food Microbiol..

[13] Zhang Y., Shen Y., Niu J., Ding F., Ren Y., Chen X., Han B.-Z. (2023). Bacteria-Induced Amino Acid Metabolism Involved in Appearance Characteristics of High-Temperature Daqu. J. Sci. Food Agric..

[14] He M., Jin Y., Zhou R., Zhao D., Zheng J., Wu C. (2022). Dynamic Succession of Microbial Community in Nongxiangxing Daqu and Microbial Roles Involved in Flavor Formation. Food Res. Int..

[15] Liu W.-H., Chai L.-J., Wang H.-M., Lu Z.-M., Zhang X.-J., Xiao C., Wang S.-T., Shen C.-H., Shi J.-S., Xu Z.-H. (2024). Community-Level Bioaugmentation Results in Enzymatic Activity- and Aroma-Enhanced Daqu through Altering Microbial Community Structure and Metabolic Function. Food Biosci..

[16] Mao J., Liu X., Gao T., Gu S., Wu Y., Zhao L., Ma J., Li X., Zhang J. (2022). Unraveling the Correlations between Bacterial Diversity, Physicochemical Properties and Bacterial Community Succession during the Fermentation of Traditional Chinese Strong-Flavor Daqu. LWT.

[17] Tong W., Li Y., Yang Y., Huang Z., Wang S., Huang D., Luo H., Zhao L. (2023). Dynamic Analysis Caffeic Acid Production Driven by the Key Physicochemical Factor and Microbial Community Succession in *Baijiu Daqu*: A Multi-Microorganism Fermentation of Solid-State Fermentation System. LWT.

[18] Zhu C., Cheng Y., Zuo Q., Huang Y., Wang L. (2022). Exploring the Impacts of Traditional Crafts on Microbial Community Succession in Jiang-Flavored Daqu. Food Res. Int..

[19] Louw N.L., Lele K., Ye R., Edwards C.B., Wolfe B.E. (2023). Microbiome Assembly in Fermented Foods. Annu. Rev. Microbiol..

[20] Huang Y., Li D., Mu Y., Zhu Z., Wu Y., Qi Q., Mu Y., Su W. (2024). Exploring the Heterogeneity of Community and Function and Correspondence of “Species-Enzymes” among Three Types of Daqu with Different Fermentation Peak-Temperature via High-Throughput Sequencing and Metagenomics. Food Res. Int..

[21] Shi W., Chai L.-J., Fang G.-Y., Mei J.-L., Lu Z.-M., Zhang X.-J., Xiao C., Wang S.-T., Shen C.-H., Shi J.-S. (2022). Spatial Heterogeneity of the Microbiome and Metabolome Profiles of High-Temperature Daqu in the Same Workshop. Food Res. Int..

[22] Tang J., Rao J., Zou Y., Liao L., Huang D., Luo H. (2023). The Community Assembly Patterns Determined Differences between the Surface and the Core Microbial Communities of Nongxiangxing Daqu. LWT.

[23] (2011). General Methods of Analysis for Daqu.

[24] (2019). Determination of Amino Acids in Feeds.

[25] Fan Q., Wang X., Zhao Y., Zheng F., Li H., Zhang F., Zhang Y., Chen F. (2019). Characterization of Key Aroma Com-pounds in Laobaigan Chinese Baijiu by GC×GC-TOF/MS and Means of Molecular Sensory Science. Flavour Fragr. J..

[26] Stegen J.C., Lin X., Konopka A.E., Fredrickson J.K. (2012). Stochastic and Deterministic Assembly Processes in Subsurface Microbial Communities. ISME J..

[27] Xun W., Li W., Xiong W., Ren Y., Liu Y., Miao Y., Xu Z., Zhang N., Shen Q., Zhang R. (2019). Diversity-Triggered Deterministic Bacterial Assembly Constrains Community Functions. Nat. Commun..

[28] Sloan W.T., Lunn M., Woodcock S., Head I.M., Nee S., Curtis T.P. (2006). Quantifying the Roles of Immigration and Chance in Shaping Prokaryote Community Structure. Environ. Microbiol..

[29] Chen W., Ren K., Isabwe A., Chen H., Liu M., Yang J. (2019). Stochastic Processes Shape Microeukaryotic Community Assembly in a Subtropical River across Wet and Dry Seasons. Microbiome.

[30] Zhang L., Xiong S., Du T., Xiao M., Peng Z., Xie M., Guan Q., Xiong T. (2023). Effects of Microbial Succession on the Dynamics of Flavor Metabolites and Physicochemical Properties during Soy Sauce Koji Making. Food Biosci..

[31] Hu X., Chen P., Tian J., Huang D., Luo H., Huang D. (2021). Predicting the Moisture Content of Daqu with Hyperspectral Imaging. Int. J. Food Eng..

[32] Yang F., Liu Y., Chen L., Li J., Wang L., Du G. (2020). Genome Sequencing and Flavor Compound Biosynthesis Pathway Analyses of Bacillus Licheniformis Isolated from Chinese Maotai-Flavor Liquor-Brewing Microbiome. Food Biotechnol..

[33] Zhou Q., Ma K., Song Y., Wang Z., Fu Z., Wang Y., Zhang X., Cui M., Tang N., Xing X. (2022). Exploring the Diversity of the Fungal Community in Chinese Traditional *Baijiu Daqu* Starters Made at Low-, Medium- and High-Temperatures. LWT.

[34] Tang P., Wang L., Zhao Q., Lu J., Qiao M., Li C., Xiao D., Guo X. (2024). Characterization of Key Aroma Compounds and Relationship between Aroma Compounds and Sensory Attributes in Different Quality of High Temperature Daqu. LWT.

[35] Deng C., Gao R., Zhao Y., Miu L., Wang M., Liu P., Chen J., Fan P. (2022). Relationship between sensory indexes, physicochemical indexes, microbial community and volatile compounds in high-temperature Daqu. Food Ferment. Ind..

[36] Shi G., Fang C., Xing S., Guo Y., Li X., Han X., Lin L., Zhang C. (2024). Heterogenetic Mechanism in High-Temperature Daqu Fermentation by Traditional Craft and Mechanical Craft: From Microbial Assembly Patterns to Metabolism Phenotypes. Food Res. Int..

[37] Zhang Y., Shen Y., Cheng W., Wang X., Xue Y., Chen X., Han B.-Z. (2021). Understanding the Shifts of Microbial Community and Metabolite Profile From Wheat to Mature Daqu. Front. Microbiol..

[38] Ren H., Sun Y., Yang Y., Li Y., Guo X., Zhang B., Zhao H., Ma D., Zhang Z. (2024). Unraveling the Correlations between Microbial Communities and Metabolic Profiles of Strong-Flavor Jinhui *Daqu* with Different Storage Periods. Food Microbiol..

[39] Zhu C., Cheng Y., Shi Q., Ge X., Yang Y., Huang Y. (2023). Metagenomic Analyses Reveal Microbial Communities and Functional Differences between *Daqu* from Seven Provinces. Food Res. Int..

[40] Liu Y., Li H., Dong S., Zhou Z., Zhang Z., Huang R., Han S., Hou J., Pan C. (2023). Dynamic Changes and Correlations of Microbial Communities, Physicochemical Properties, and Volatile Metabolites during Daqu Fermentation of Taorong-Type Baijiu. LWT.

[41] Zhu M., Zheng J., Xie J., Zhao D., Qiao Z.-W., Huang D., Luo H.-B. (2022). Effects of Environmental Factors on the Microbial Community Changes during Medium-High Temperature Daqu Manufacturing. Food Res. Int..

[42] Wu M., Luo Y., Yao Y., Ji W., Xia X. (2024). Multidimensional Analysis of Wheat Original Crucial Endogenous Enzymes Driving Microbial Communities Metabolism during High-Temperature Daqu Fermentation. Int. J. Food Microbiol..

[43] Stegen J.C., Lin X., Fredrickson J.K., Chen X., Kennedy D.W., Murray C.J., Rockhold M.L., Konopka A. (2013). Quantifying Community Assembly Processes and Identifying Features That Impose Them. ISME J..

[44] Evans S., Martiny J.B.H., Allison S.D. (2017). Effects of Dispersal and Selection on Stochastic Assembly in Microbial Communities. ISME J..

[45] Yang L., Ning D., Yang Y., He N., Li X., Cornell C.R., Bates C.T., Filimonenko E., Kuzyakov Y., Zhou J. (2022). Precipitation Balances Deterministic and Stochastic Processes of Bacterial Community Assembly in Grassland Soils. Soil Biol. Biochem..

[46] Ramoneda J., Jensen T.B.N., Price M.N., Casamayor E.O., Fierer N. (2023). Taxonomic and Environmental Distribution of Bacterial Amino Acid Auxotrophies. Nat. Commun..

[47] Devendran S., Shrestha R., Alves J.M.P., Wolf P.G., Ly L., Hernandez A.G., Méndez-García C., Inboden A., Wiley J., Paul O. (2019). Clostridium Scindens ATCC 35704: Integration of Nutritional Requirements, the Complete Genome Sequence, and Global Transcriptional Responses to Bile Acids. Appl. Environ. Microbiol..

[48] Veith N., Solheim M., van Grinsven K.W.A., Olivier B.G., Levering J., Grosseholz R., Hugenholtz J., Holo H., Nes I., Teusink B. (2015). Using a Genome-Scale Metabolic Model of Enterococcus Faecalis V583 To Assess Amino Acid Uptake and Its Impact on Central Metabolism. Appl. Environ. Microbiol..

[49] Walzem R.L., Dillard C.J., German J.B. (2002). Whey Components: Millennia of Evolution Create Functionalities for Mammalian Nutrition: What We Know and What We May Be Overlooking. Crit. Rev. Food Sci. Nutr..

[50] Mee M.T., Collins J.J., Church G.M., Wang H.H. (2014). Syntrophic Exchange in Synthetic Microbial Communities. Proc. Natl. Acad. Sci. USA.

[51] Yuan S., Du H., Zhao D., Qiao Z., Zheng J., Yu X., Xu Y. (2023). Stochastic Processes Drive the Assembly and Metabolite Profiles of Keystone Taxa during Chinese Strong-Flavor Baijiu Fermentation. Microbiol. Spectr..

[52] Deng Y., Jiang Y.-H., Yang Y., He Z., Luo F., Zhou J. (2012). Molecular Ecological Network Analyses. BMC Bioinform..

[53] Yuan M.M., Guo X., Wu L., Zhang Y., Xiao N., Ning D., Shi Z., Zhou X., Wu L., Yang Y. (2021). Climate Warming Enhances Microbial Network Complexity and Stability. Nat. Clim. Change.

[54] Shi S., Nuccio E.E., Shi Z.J., He Z., Zhou J., Firestone M.K. (2016). The Interconnected Rhizosphere: High Network Complexity Dominates Rhizosphere Assemblages. Ecol. Lett..

[55] Xun W., Liu Y., Li W., Ren Y., Xiong W., Xu Z., Zhang N., Miao Y., Shen Q., Zhang R. (2021). Specialized Metabolic Functions of Keystone Taxa Sustain Soil Microbiome Stability. Microbiome.

[56] Coyte K.Z., Schluter J., Foster K.R. (2015). The Ecology of the Microbiome: Networks, Competition, and Stability. Science.

[57] Fontaine C., Guimarães Jr P.R., Kéfi S., Loeuille N., Memmott J., van der Putten W.H., van Veen F.J.F., Thébault E. (2011). The Ecological and Evolutionary Implications of Merging Different Types of Networks. Ecol. Lett..

[58] Yang L., Fan W., Xu Y. (2021). GC × GC-TOF/MS and UPLC-Q-TOF/MS Based Untargeted Metabolomics Coupled with Physicochemical Properties to Reveal the Characteristics of Different Type Daqus for Making Soy Sauce Aroma and Flavor Type Baijiu. LWT.

[59] Liu J., Zhao W., Zhang A., Li P., Liu J. (2024). Dynamics and Functionalities of Bacterial Community during Foxtail Millet Dough Fermentation by Metagenomic Analysis. J. Future Foods.

[60] Ortiz M., Leung P.M., Shelley G., Jirapanjawat T., Nauer P.A., Van Goethem M.W., Bay S.K., Islam Z.F., Jordaan K., Vikram S. (2021). Multiple Energy Sources and Metabolic Strategies Sustain Microbial Diversity in Antarctic Desert Soils. Proc. Natl. Acad. Sci. USA.

